# *Drosophila melanogaster* Selection for Survival after Infection with *Bacillus cereus* Spores: Evolutionary Genetic and Phenotypic Investigations of Respiration and Movement

**DOI:** 10.1155/2013/576452

**Published:** 2013-03-21

**Authors:** Junjie Ma, Andrew K. Benson, Stephen D. Kachman, Deidra J. Jacobsen, Lawrence G. Harshman

**Affiliations:** ^1^Department of Food Science and Technology, University of Nebraska-Lincoln, Lincoln, NE 68583, USA; ^2^Department of Statistics, University of Nebrasksa-Lincoln, Lincoln, NE 68583, USA; ^3^Department of Biology, Indiana University, Bloomington, IN, USA; ^4^School of Biological Sciences, University of Nebraska-Lincoln, Lincoln, NE 68588, USA

## Abstract

Laboratory populations of * D. melanogaster* have been subjected to selection for survival after live spores of * B. cereus* were introduced as a pathogenic agent. The present study was designed to investigate correlated traits: respiration as a metabolic trait and movement as a behavioral trait. An underlying hypothesis was that the evolution of increased survival after *B. cereus* infection exerts a metabolic cost associated with elevated immunity and this would be detected by increased respiration rates. There was support for this hypothesis in the male response to selection, but not for selected-line females. Two phenotypic effects were also observed in the study. Females especially showed a marked increase in respiration after mating compared to the other assay stages regardless of whether respiration was measured per fly or adjusted by lean mass or dry weight. Given that mating stimulates egg production, it is feasible that elevated metabolism was needed to provision oocytes with yolk. Females also moved less than males, perhaps due to behaviors related to oviposition whereas elevated male activity might be due to behaviors associated with seeking females and courtship. Relatively low movement of females indicated that their elevated respiration after mating was not due to a change in locomotion.

## 1. Introduction

 A tradeoff between immune function and reproduction has been observed in a range of studies. For example, an increase in reproductive effort is correlated with increased parasite incidence and disease [1, 2]. More generally, an increase in reproduction is associated with decreased immune system function [3, 4]. Two mechanisms have been proposed for this relationship. One is the Y model [5] in which there is an energetic competition between somatic function (immunity in the present study) and reproduction. The arms of the Y represent competition for energy between traits and the long axis represents resource input. Another potential mechanism for a tradeoff is a negative pleiotropic effect of hormones acting on two traits. An example is the effect of insulin signaling as a stimulator of reproduction and a suppressor of life span in *D. melanogaster* [6, 7]. In support of the role of hormones on insect life history tradeoffs, juvenile hormone (JH) has been shown to have negative pleiotropic effects on immunity and reproduction. In the flour beetle, *Tenebrio molitor*, mating causes a decrease in an immune system enzyme (phenoloxidase) by increasing the level of JH [8]. In the bee, *Apis mellifera*, a caste behavior was experimentally altered which increased JH and decreased both immune function and life span [9, 10]. 

 Invertebrates are useful for investigating trade-offs between immune function and life history traits [11]. A study of the ground cricket, *Allonemobius socius*, documented that an elevated number of matings was correlated with decreased immune function and life span [12]. Male *D. melanogaster* lose immune function when they mate more frequently [13]. The cost of immune function could also impact physiological or behavioral attributes which are the traits that are the focus of the research reported here.

 Studies on vertebrates are also relevant to the present study as there has been more emphasis on measuring the energetic cost of mounting an immune response in this group of organisms. In the case of a study on a sparrow (*Parus caeruleus*), the energetic cost of the antibody reaction was approximately 8% to 13% of the baseline metabolic rate [14]. However, some studies on birds have failed to detect a cost of an immune response [15] and it has been argued that the energetic cost might be low [15, 16].

There have been fewer studies on invertebrates designed to measure the cost of immune responses. However, one study is noteworthy [17]. Using bumblebee workers (*Bombus terrestris*), two elicitors of immune responses were used: lipopolysaccharides (LPS) and microlatex beads that were about the same size as bacteria. Both induced immune responses that could have been in different pathways. The cost was evaluated in terms of starvation survival time, which was decreased from 1.5 to 1.7.  Elevated lipid levels are associated with higher levels of stress resistance in selection experiments [18] and it is feasible that the immune responses in these bees depleted stored lipid for the additional metabolism needed for the immune responses. This could be the mechanism underlying decreased starvation resistance in *Bombus terrestris* as a result of induced immune responses. 

 The present study addresses the relationship between immune function and metabolism (respiration) or behavior (movement) in the context of a laboratory selection experiment on *D. melanogaster*. Lines of flies were selected for increased survival after *B. cereus* spore infection [19]. A key goal was to determine if there was a metabolic cost associated with elevated immunity in the selected lines. This cost would be inferred from increased respiration in the selected lines. Movement was also measured to determine if heightened immune function also altered activity. The questions addressed by this study are pertinent to understanding the cost of immune function, a fundamental issue in evolutionary biology [20]. 

 The present study was conducted after 19 generations of selection on *D. melanogaster*. Selection resulted in a 3-log increase in the number of spores required for 50% mortality [19]. There were nine lines: three lines were selected by puncturing the live *B. cereus* into the body at a level of spore concentration that yielded approximately 50% *D. melanogaster *mortality, three lines in which the flies were punctured with sterile water (wound control lines), and three lines that were not punctured [19]. The latter were no-perturbation control lines. 

 In the present study, hypotheses related to a metabolic cost of immunity were tested. In terms of respiration, elevated CO_2_ and O_2_ rates in the untreated selected lines would be interpreted as a metabolic cost. Increased respiration (metabolism) results from increased use of endogenous resources and this would be a cost of immunity. We also hypothesized that movement could be affected by selection for *D. melanogaster* survival after infection with *B. cereus* spores or induction of the immune response by autoclaved spores. The inclusion of movement measurements in this study potentially allowed us to assess locomotion as a factor contributing to respiration rate differences between line types and treatments.

## 2. Materials and Methods

### 2.1. *Drosophila melanogaster* Lines and Treatments

The founding of the base population used for selection, the process of selection, and the response to selection are described in Ma et al. [19]. A brief description of selection follows. There were three selected lines which were independent subpopulations originated from the base population. At each generation of selection, 1000 females and 1000 males were exposed to spores of *B. cereus*, a species that is closely related to *B. anthracis*. Spores of *B. anthracis* have been used as bioterrorism agents and an ultimate goal of the selection experiment was to identify spore resistance genes in *D. melanogaster*. The spores were introduced by puncturing them into the thorax with a fine needle. The level of selection was adjusted incrementally to obtain 50% mortality each generation. After 19 generations, there was a 3-log increase in the number of spores required for 50% mortality. Three additional lines were established as wound control lines. Flies in these lines were punctured with sterile H_2_O each generation. Three more lines were established; the flies in these lines were not perturbed (no treatment) each generation. In this study, there are line types (selected, wound control, and no-perturbation lines) treatments (punctured with autoclaved spores, punctured with water, and no treatment). The lines allow for assessment of genetic correlations and the treatments allow for assessment of phenotypic effects. The designation and description of lines and treatments and the successive stages of the respiration assay are shown in Table 1. “Line types” refers to the set of selected lines and the control lines (wound-control or no perturbation). 

 Prior to assays, flies from the selection and control lines were handled in a defined manner that is described below. Before conducting any of the assays, the selection experiment was relaxed (no selection) for two generations. To relax selection, all lines were reared without exposure to spores or wounding. It is important to emphasize that at this point there has been no explicit selection for two generations as all lines were reared in the same unperturbed manner. Each rearing vial was seeded with 100 eggs to standardize density. Flies were raised on a cornmeal, molasses, torula yeast Drosophila food [19]. Prior to respiration or movement assays, flies were maintained in a room with 12-hour light-dark cycle at 25°C. In this environment, males and females from all lines were exposed to one of the following three conditions which are related to selection and control line environments: introduction of autoclaved (dead) spores with a tungsten needle (to induce an immune response) or punctured with a needle dipped in sterile H_2_O (to induce a response to wounding) or left untreated. After three days in the room with a 12-hour light/dark cycle at 25°C, respiration assays were initiated. A movement study was conducted on the same flies after the respiration assay. The flies were 3–7 days old at the time of the respiration assays and 8 days old at the time of the movement assay. 

### 2.2. Bacterial Culture and Spore Isolation

*Bacillus cereus* ATCC 10987 was used as a source of spores. Spore purification was conducted using a step gradient of Renografin [21]. Biomass for gradient purification was generated from a single colony of *B. cereus* grown in 25 mL of Difco sporulation medium and incubated at 37°C on a rotary shaker (150 rpm) until mid-log phase. This culture was expanded into 2 L of DSM from a 1 : 10 dilution, followed by incubation at 37°C on the rotary shaker for 48 hours. The pellet was resuspended in 200 mL of sterile water and stored overnight at 4°C. The pellet was resuspended in 200 mL of 4°C sterile water followed by the same centrifugation procedure. The pellet was again resuspended in 200 mL of sterile water and stored overnight at 4°C. After repeating the centrifuge-resuspension-centrifugation procedure, greater than 90% bright-field spores were observed by phase contrast microscopy. The spores and cell debris were harvested one final time and the pellet was resuspended in 20% Renografin then transferred to a 30 mL glass core tube with 15 mL of 50% Renografin. The spore suspension was centrifuged for 30 min at 4°C at 10,000 ×g. All layers containing vegetative cells were removed and the spore pellet retained. The pellet was resuspended in 10 mL of 4°C sterile water in an Oak Ridge tube. The spore suspension was centrifuged for 10 min at 10,000 ×g at 4°C. Trace amounts of Renografin were removed by 3 washes with 4°C sterile water as described above. The spore pellet was suspended in 2 ml 4°C sterile water. The concentration of spores was determined by serial dilution and spread plating.

 Because of the need for increasing amounts of spores during selection, spores were prepared on two different occasions during selection (one preparation used for selection generations 1–11 and the second preparation used for selection generations 12–19). Each preparation was normalized for concentration and the normalized preparations resulted in very similar LD50s on the selected lines. The second preparation was the source of autoclaved spores used in this study. 

### 2.3. Respiration

The experimental subjects for respiration assays consisted of four cohorts of flies which were all from generation 19. Temporally separated cohorts allowed for all the subjects to be assayed within the time available after selection generation 19. Temporal variation between cohorts was achieved by adjusting the four times of egg collection to create a timed-gap between cohorts. Respiration was measured on adult flies at three successive assay stages which are denoted as “day 3” (before treatment), “day 5” (after puncture with autoclaved spores or sterile H_2_O or remained untreated), or “day 7” (after mating) (Table 1). This progression mimics the successive steps of the selection process. For example, 24 hours of mating was conducted prior to the last stage (“day 7” posteclosion, Table 1) in the respiration assay similar to the process of breeding in the selected and control lines to produce the next generation.

 All respiration assays were conducted in a controlled manner. These assays were conducted during the 12-hour light period to eliminate behavior changes caused by a change of light. All flies used for the respiration assay were held in syringes that were otherwise empty when respiration measurements were taken. These flies were postprandial. There was no opportunity for artifacts arising from flies using dietary sources during the process of respiration measurement. The flies were only apart from food during the time that respiration was measured. The maximum time away from food was 19 minutes and thus there was no desiccation stress. The samples in one set (all permutations of lines, treatments and sex) were temporally randomized within the 12-hour light-cycle period. One set, (a replicate) of all sample types, was conducted in one 12-hour period. For each of the four cohorts (described in the first paragraph of this section), three replicates were run for each type of sample consisting of assay stages, line types, and sexes. 

 Respiration of the flies was measured using a parallel stop flow system and injecting air sampled from fly vials into a flow-through respirometry setup. Water-scrubbed ambient air (Drierite/soda lime) was used as the carrier gas in the flow-through system. A Nalgene carboy was used to remove short-term fluctuations in gas concentrations before the scrubber column. CO_2_ was measured using an infrared gas analyzer (CA-10a, Sable Systems International, Las Vegas, NV, USA). O_2_ was measured using a fuel cell-based oxygen analyzer (FC-10a, Sable Systems International, Las Vegas, NV, USA). The air flow was adjusted to 50 to 80 mL/min. Details about the mechanics, process, and calculations used for respiration can be found in Lighton [22]. 

 For any one sample, respiration was measured using three flies that were aspirated into a 5 mL plastic syringe. The syringe had a three-way valve that closed the interior to outside air and gases accumulated. Before the respiration measurements, syringes were held within a PELT-5 environmental chamber at 25°C in the light for at least seven minutes. At the end of this period, each syringe was attached to a tubing port and opened to allow gas to be injected into the respiration instrumentation. A standard volume of almost all of the air in the syringe was introduced into the tubing leading to the CO_2_ and O_2_ monitors in succession. For a base line, a blank syringe with only scrubbed air was used for each set of flies subjected to respirometry. After respirometry, the ambient air within each syringe was flushed and replaced by the scrubbed air prior to introduction of the next set of flies. The respiration measurements were normalized (divided) by the length of time that flies were in syringes. 

 Respiration rates were adjusted by lean mass or dry weight for each line, treatment, and sex. The mean values for respiration were divided by the mean values for lean mass or dry weight. The mean values and standard errors were derived from the three replicate lines of each type.

### 2.4. Movement

 After each respiration rate measurement, the same flies were used for a movement assay. The flies were 8 days posteclosion which was one day older than the postmating assay stage. The number of movements of an individual fly was recorded every 10 minutes for 24 hours. There were two monitors (TriKinetics) with a total of 64 tubes to measure movement. For each measurement, an individual fly was aspirated into a glass capillary testing tube (5 mm in diameter and 65 mm in length). One end of the testing tube was partially covered with fly food so that flies would not be subjected to starvation, given the length of the assay. Moreover, the presence of food more closely replicates conditions the flies were exposed to in the selection experiment. A hole in the food plug allowed air to enter the tube. The other end of each tube was inserted into a placement site in the instrument. The location of each sample among and within the monitors was randomized to avoid bias potentially associated with position. Movement was recorded when a fly interfered with a laser beam projected through the set of tubes in a monitor. Two damp cotton balls were placed in a plastic bag surrounding each movement measurement device to maintain relatively high humidity. The assay was conducted at 25°C with a 12 : 12 light : dark cycle as this was the environment flies were held prior to assays.

### 2.5. Weight

 Flies from generation selection generation 36 were used for weight measurements. As always was the case, selection was relaxed for two generations prior to weight measurements. After freezing for dry weight measurement, each fly was placed in an otherwise empty 2 mL eppendorf tube. The cap was left open and the flies were placed in a drying oven (70°C) with air blown throughout the chamber. The flies were left to dry for 24 hours. The weight was taken on individual flies and 10 flies were weighted for each treatment, sex, line, and assay stage. A Sartorius microbalance (M2P) was used for all fly weights. After the dry weight of flies was obtained, the flies were placed individually in 2 ml eppendorf tubes with 2 mL of ethyl ether. The caps were closed and the tubes were shaken for 24 hours to extract lipids. The flies were again dried and reweighed to obtain lean mass measurements. The conditions used for the weight measurements paralleled the conditions used for respiration assays.

### 2.6. Data Analysis

The data were analyzed by repeated measures ANOVA, ANOVA, and the Tukey-Kramer test using SAS version 9.3. Mixed model ANOVAs for each sex were employed with line types and treatments as fixed effects; the variations among lines of the same type were the random effects. Only CO_2_ measures were used for statistical analysis. The O_2_ values and trends were essentially the same as CO_2_ as can be seen from the fact that respiration quotients are close to 1.00 for all treatments and lines.

## 3. Results

### 3.1. Statistical Analyses

 Introducing line-to-line random effects improved the fit of the model in the analysis of CO_2_ respiration rate adjusted by the lean mass or dry weight for both males and females, and CO_2_ respiration rate measured per fly for females (see Table S1A in Supplementary Material available online at http://dx.doi.org/10.1155/2013/576452). Therefore, this source of variation was incorporated in the analysis. However, adding line-to-line random effects did not improve the fit of the model when respiration rate was measured per fly for males. All of the *P* values for respiration rates and weights and post hoc analyses of respiration rates are reported in supplementary tables (see Tables S1B, S1C and S1D in Supplementary Material). 

### 3.2. Respiration 

#### 3.2.1. Line Types and Treatments

**Figure 1:**
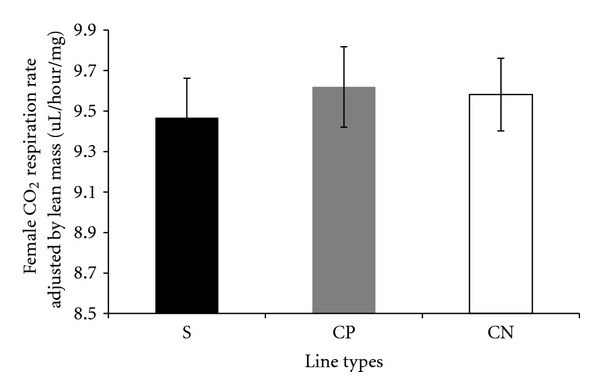
Average CO_2_ respiration rates adjusted by lean mass for females from selected and control lines. S: selected lines, CP: wound control lines, CN: no perturbation control lines.

**Figure 2:**
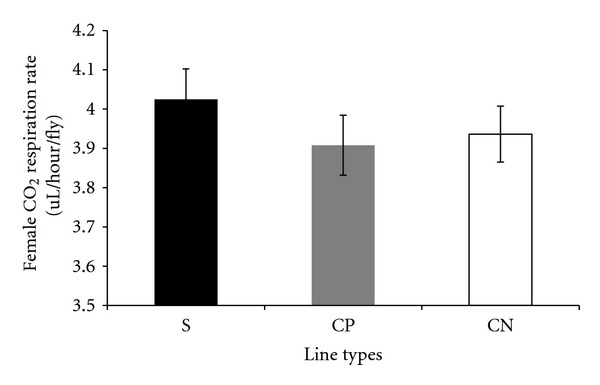
Average CO_2_ respiration rates measured per fly for females from selected and control lines. S: selected lines, CP: wound control lines, CN: no perturbation control lines.

**Figure 3:**
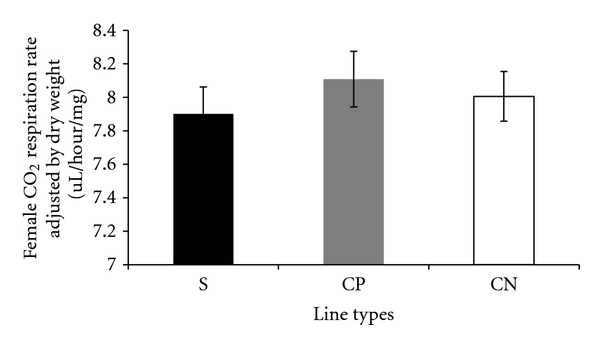
Average CO_2_ respiration rates adjusted by dry weight for females from selected and control lines. S: selected lines, CP: wound control lines, CN: no perturbation control lines.

**Figure 4:**
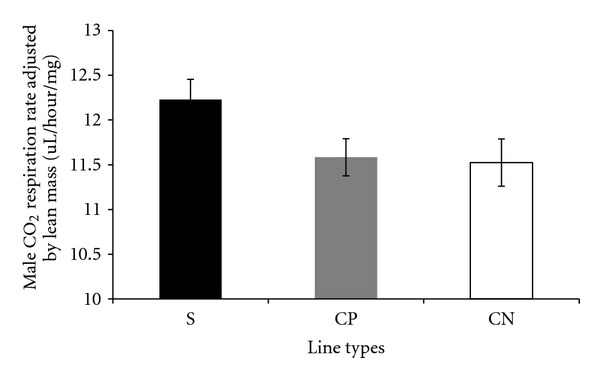
Average CO_2_ respiration rates adjusted by lean mass for males from selected and control lines. S: selected lines, CP: wound control lines, CN: no perturbation control lines.

**Figure 5:**
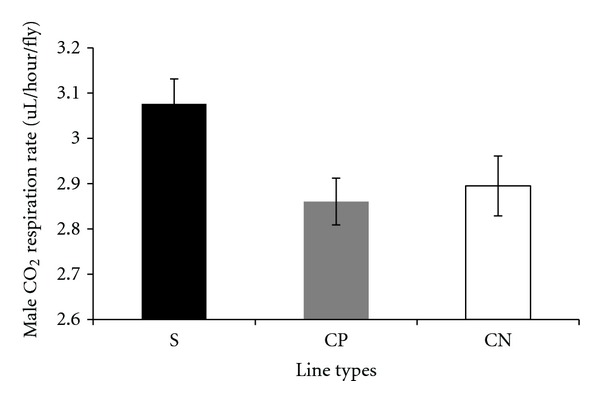
Average CO_2_ respiration rates measured per fly for males from selected and control lines. S: selected lines, CP: wound control lines, CN: no perturbation control lines.

**Figure 6:**
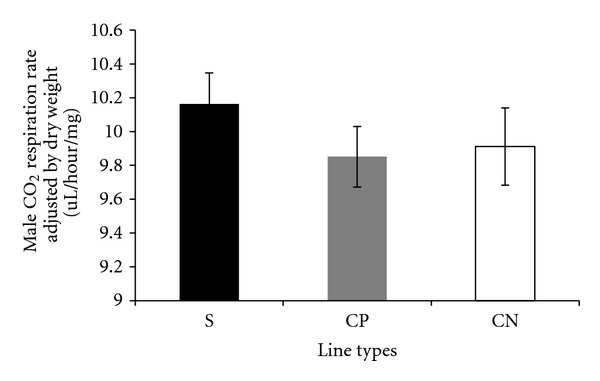
Average CO_2_ respiration rates adjusted by dry weight for males from selected and control lines. S: selected lines, CP: wound control lines, CN: no perturbation control lines.

 Carbon dioxide respiration rates adjusted by lean mass, measured per fly, and adjusted by dry weight in the selected and control lines are presented in a series of figures (female: Figures 1, 2, and 3; male: Figures 4, 5, and 6). The numerical values for average respiration rates in the present study are presented in supplementary tables (see Tables S4A, S4B, and S4C). There were no statistically significant differences among line types or treatments for females or males when respiration was adjusted by lean mass or adjusted by dry weight. However, there was a sex by line type interaction when respiration rates were adjusted by lean mass (*P* = 0.0513). Underlying this interaction, males exhibited an elevated respiration rate in the selected lines (compared to females); male respiration rates were lower in the CP and CN lines whereas female rates were almost invariant among line types (Figure 19). For females, respiration rates per fly were not significantly different among line types or treatments. For males, there was a statistically significant difference among line types (*P* = 0.0132, see Table S1B). The pairwise post hoc statistical analysis based on comparisons of per fly measurements between line types indicated that the selected line males had higher level of respiration than the punctured control lines (*P* = 0.0186) and the no perturbation control lines (*P* = 0.0494). There was no statistically significant difference between the two types of control lines (*P* = 0.9303). 

#### 3.2.2. Assay Stages

**Figure 7:**
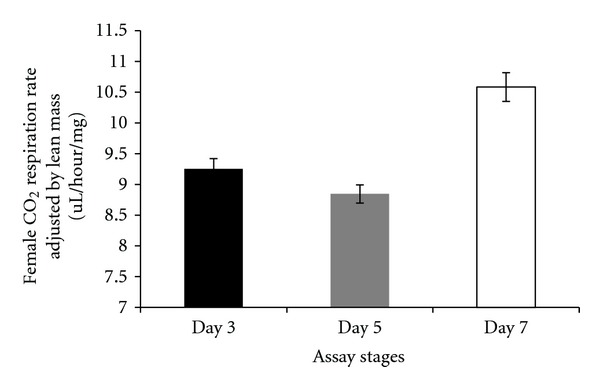
Average CO_2_ respiration rates adjusted by lean mass for females at different assay stages. Day 3: before perturbations, Day 5: punctured with autoclaved spores or punctured with sterile H_2_O or remained unperturbed, Day 7: after mating.

**Figure 8:**
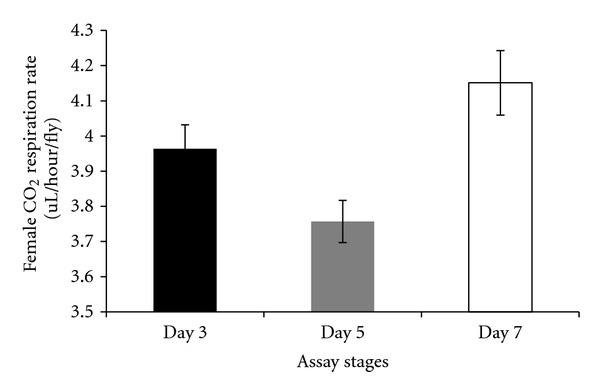
Average CO_2_ respiration rates measured per fly for females at different assay stages. Day 3: before perturbations, Day 5: punctured with autoclaved spores or punctured with sterile H_2_O or remained unperturbed, Day 7: after mating.

**Figure 9:**
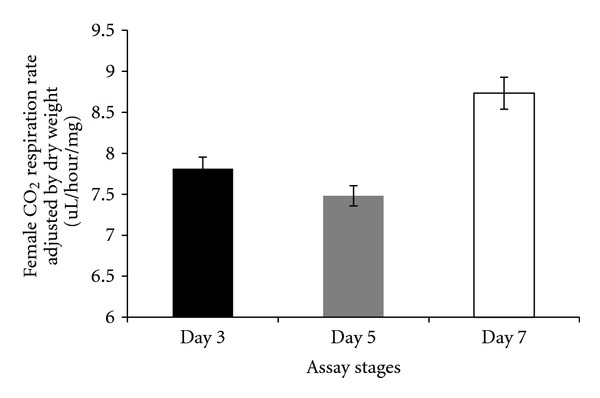
Average CO_2_ respiration rates adjusted by dry weight for females at different assay stages. Day 3: before perturbations, Day 5: punctured with autoclaved spores or punctured with sterile H_2_O or remained unperturbed, Day 7: after mating.

**Figure 10:**
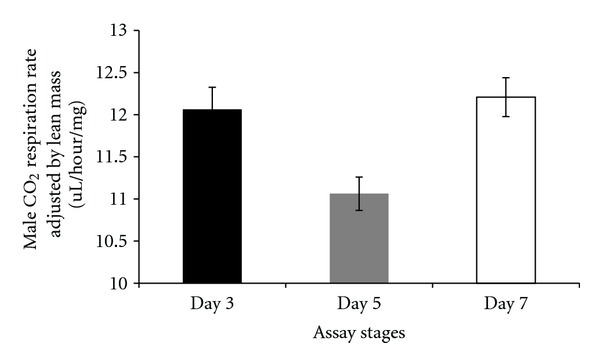
Average CO_2_ respiration rates adjusted by lean mass for males at different assay stages. Day 3: before perturbations, Day 5: punctured with autoclaved spores or punctured with sterile H_2_O or remained unperturbed, Day 7: after mating.

**Figure 11:**
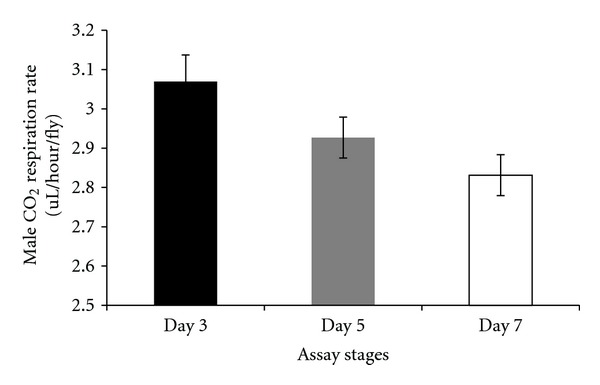
Average CO_2_ respiration rates measured per fly for males at different assay stages. Day 3: before perturbations, Day 5: punctured with autoclaved spores or punctured with sterile H_2_O or remained unperturbed, Day 7: after mating.

**Figure 12:**
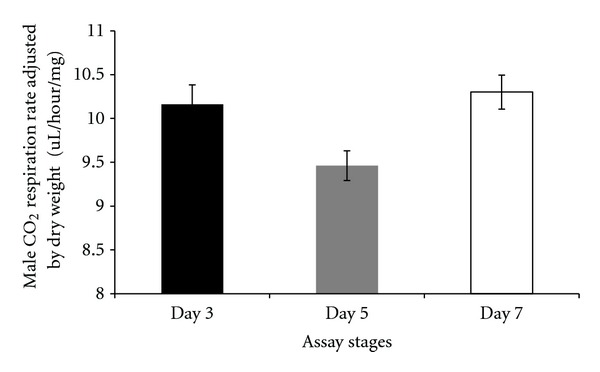
Average CO_2_ respiration rates adjusted by dry weight for males at different assay stages. Day 3: before perturbations, Day 5: punctured with autoclaved spores or punctured with sterile H_2_O or remained unperturbed, Day 7: after mating.

There were differences in respiration rates among assay stages for females and males. The average CO_2_ release rate of females was markedly higher after mating (day 7) and lowest at day 5 (females: Figures 7, 8, and 9). Males showed the same pattern as females except for the data determined per fly. In this case, the average CO_2_ respiration rate was the lowest after mating (day 7), but the highest before treatment (day 3) (males: Figures 10, 11, and 12). Overall for females, there were statistically significant differences when respiration was adjusted by lean mass (*P* < 0.0001) or dry weight (*P* < 0.0001), or per fly (*P* < 0.0001). Overall for males, there were statistically significant differences when respiration was adjusted by lean mass (*P* = 0.0005) or dry weight (*P* = 0.0046), or measured per fly (*P* < 0.0087). Post hoc analysis by Tukey's method showed that there were statistically significant differences among assay stages. Post hoc comparisons for females when respiration was adjusted by lean mass indicated that respiration was the highest after mating (day 7) compared to day 5 (*P* < 0.0001) and day 7 compared to day 3 (*P* < 0.0001). The same pattern of statistically significant differences was observed when female respiration data was adjusted by dry weight. For female respiration per fly, the only statistically significant post hoc difference was between day 7 after mating and day 5 after treatment (*P* < 0.0001). Post hoc comparisons for males when respiration was adjusted by lean mass indicated that respiration was the lowest at day 5 after treatment compared to day 3 (*P* = 0.0044) or day 7 (*P* = 0.0011). When male respiration rates were adjusted by dry weight, day 5 respiration rates were the lowest compared to day 3 (*P* = 0.0250) and day 7 (*P* = 0.0067). For male respiration rates measured per fly, the respiration rate was the lowest on day 7 after mating which was statistically significantly different than day 3 before treatment (*P* = 0.0064).

**Figure 13:**
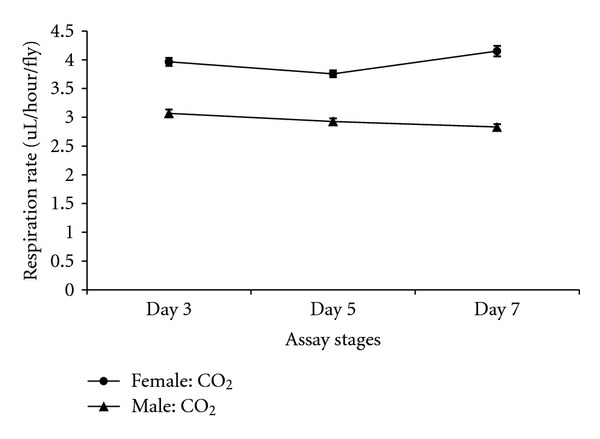
Line graph of average CO_2_ respiration rates measured per fly for females and males at different assay stages. Day 3: before perturbations, Day 5: punctured with autoclaved spores or punctured with sterile H_2_O or remained unperturbed, Day 7: after mating.

**Figure 14:**
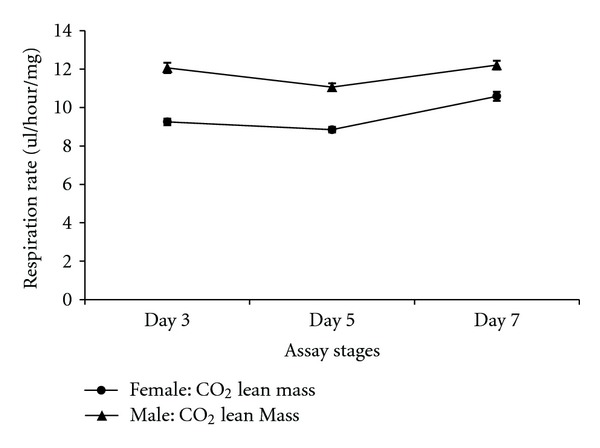
Line graph of average CO_2_ respiration rates adjusted by lean mass for females and males at different assay stages. Day 3: before perturbations, Day 5: punctured with autoclaved spores or punctured with sterile H_2_O or remained unperturbed, Day 7: after mating.

 There were statistically significant interactions between sex and the three assay stages for CO_2_ respiration rates. When respiration rates were measured per fly, there was a statistically significant interaction between sex and assay stages (*P* < 0.0001). For females when respiration was measured per fly, the highest CO_2_ respiration rates were observed at the after-mating assay stage (day 7) while the lowest were at day 5 (Figure 13). On the other hand, for males measured per fly the highest CO_2_ respiration rate was observed at the day 3 assay stage and the lowest was present at the postmating stage (Figure 13). There was also statistical support for an interaction between sex and assay stages when respiration rate data were adjusted by lean mass (*P* = 0.0095). Both females and males showed the same pattern, in which the postmating assay stage (day 7) had the highest CO_2_ respiration rate while posttreatment assay stage (day 5) had the lowest. The increase in respiration rate on day 7 was greater for females than males (Figure 14) contributing to the significant interaction between assay stages and sexes. 

 Respiration rates (adjusted by lean mass or per fly) are shown for each line within a line type for females and males (see Figures S2A, S2B, S2C, and S2D). For females, the selected line means are not consistently higher for selected lines versus control-punctured and no perturbation control lines. For males, the line means of respiration rates are consistently higher for selected lines when measured per fly (S2D), but not when adjusted by lean mass (S2B).

#### 3.2.3. Respiratory Quotients

 Respiratory quotients were calculated from the data as described in Section 2. All RQ values were close to 1.0 (females Table 2(a), males Table 2(b)) which indicated that carbohydrates were used for respiration. 

### 3.3. Movement

**Figure 15:**
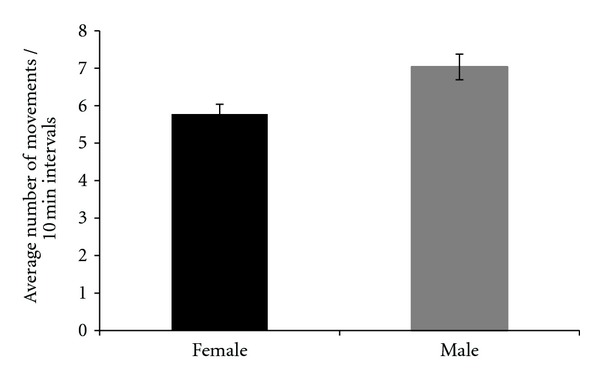
Average number of movements of females and males in 10-minute intervals detected when an individual interrupted the path of a laser beam in a movement monitor. Each assay was conducted for 24 hours.

Movement for both sexes was investigated for all of the line types and treatments. The statistical analysis of female movement data indicated no significant line or treatment effects. The statistical analysis of male movement data indicated that there was no significant effect of line type or treatment effect. The overall analysis of the data set revealed a significant difference between sexes (*P* = 0.011); males moved more frequently than females (Figure 15). 

### 3.4. Weight

**Figure 16:**
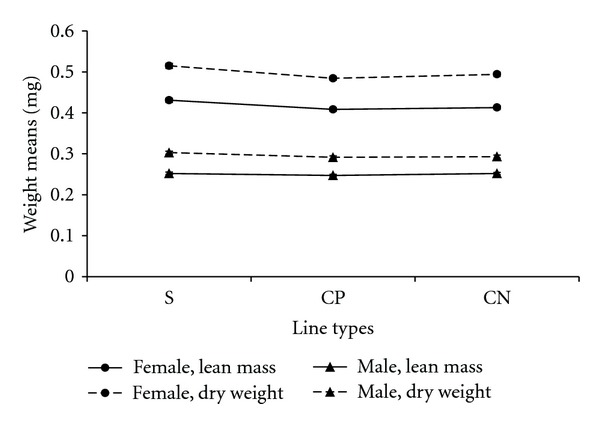
Weight (dry weight, lean mass) means for females and males from different line types. S: selected lines, CP: wound control lines, CN: no perturbation control lines.

Figure 16 shows the dry weight and lean mass data for each line type and sex. There was a statistically significant difference among line types for female lean mass (*P* = 0.0004) and female dry weight (*P* < 0.0001). There was a statistically significant difference among line types for male dry weight (*P* = 0.0334), but not for lean mass (*P* = 0.3050). The difference between dry weight and lean mass is mostly due to neutral lipids (mainly triacylglycerides and diglycerides) that were extracted from dry weight flies to generate the flies that were used for lean mass measurements (see Section 4 for relevance to respiration rates). 

**Figure 17:**
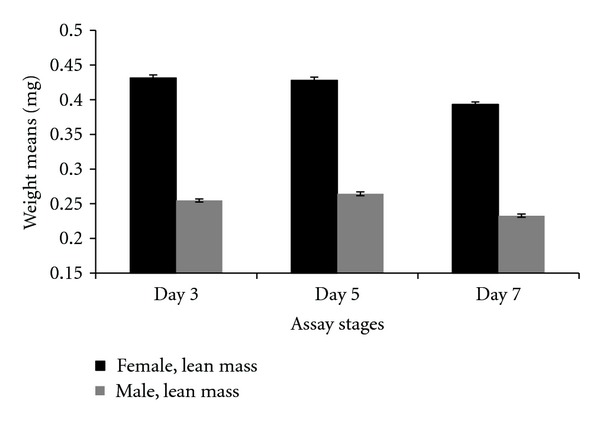
Weight (lean mass) means for females and males at different assay stages. Day 3—before perturbations, Day 5: punctured with autoclaved spores or punctured with sterile H_2_O or remained unperturbed, Day 7: after mating.

**Figure 18:**
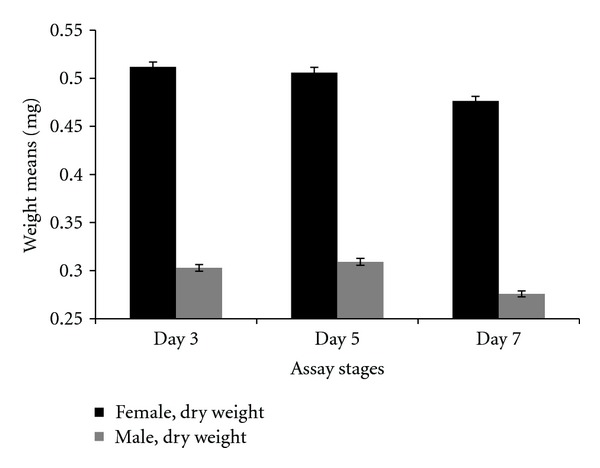
Weight (dry weight) means for females and males at different assay stages. Day 3: before perturbations, Day 5: punctured with autoclaved spores or punctured with sterile H_2_O or remained unperturbed, Day 7: after mating.

 A higher level of lipid was present in females than males. This was inferred by the observation that females exhibited a greater drop from dry weight to lean mass than males (see Figure S3). This contributed to a statistically significant interaction between weights (dry weight and lean mass) and sex (*P* < 0.0001). Both males and females lost weight (dry weight or lean mass) after mating (Figures 17 and 18). This was shown by the post hoc statistical analysis for females and males comparing the posttreatment assay stage (day 5) and postmating assay stage (day 7). For the female comparison of these two assay stages, the *P* value was *P* < 0.0001 for lean mass and *P* = 0.0010 for dry weight. For males the *P* values were *P* < 0.0001 for both lean mass and dry weight. 

## 4. Discussion

### 4.1. Overview

**Figure 19:**
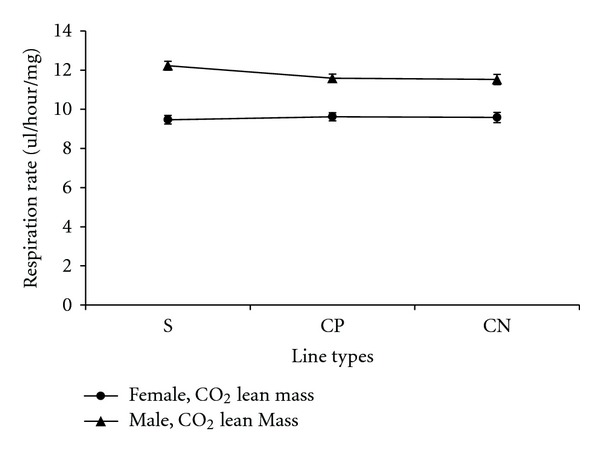
Line graph of average CO_2_ respiration rates adjusted by lean mass for females and males among different line types. S: selected lines, CP: wound control lines, CN: no perturbation control lines.

 There were a number of traits investigated in this study that were associated with sex differences. There was mixed evidence that selected-line males exhibited an indirect response to selection that resulted in a higher level of CO_2_ respiration rates (O_2_ respiration rates exhibited a parallel pattern). There was consistently statistically significant support for this relationship when respiration rate was measured per fly. When respiration rate was adjusted by lean mass, the level of support for an interaction between sex and line types approached statistical significance; the *P* value is reported in the first paragraph of Section 2.3. Overall, there was evidence that selected-line males exhibited higher respiration rates (Figures 4, 5, and 19) whereas selected-line females did not (Figures 1, 2, and 19), but the data and statistical analyses did not uniformly support this observation as discussed detail in the following section of the Discussion. There were marked differences in respiration rate between assay stages. For females, when respiration was measured per fly or adjusted by lean mass or dry weight, respiration rates were the lowest at day 5 and markedly higher after mating (day 7). The high rate of female respiration after mating could be due to an increase in the synthesis of protein and lipid needed to provision the oocytes with yolk (vitellogenesis). Finally, there was a difference between males and females in movement. Hypothetically, males were more active as a result of mate-seeking behaviors versus females who were relatively stationary reflecting oviposition-related behaviors. 

### 4.2. Respiration Rates

 The hypothesis underlying this study was that evolved resistance to *B. cereus* spores was due to a physiologically costly immune system response to selection that would be reflected in elevated respiration rates. Elevated respiration was observed in selected-line males when the respiration rate was determined per fly. Also, there was an indication of an elevated male respiration rate from the interaction between sex and lines types when data was adjusted by lean mass. Respiration was elevated in selected-line males compared to the other line types whereas this was not observed for females (Figure 19). The observations and data analyses described above in this paragraph suggested a *D. melanogaster* cost associated with selection for survival after *B. cereus* spore infection. On the other hand, there were no main-effect differences in male respiration rates adjusted by lean mass. Post hoc tests were nevertheless conducted and there were no statistically significant pairwise differences between line types in male respiration rate adjusted by lean mass. Thus, the evidence for an increase in male respiration rates in the selected lines was mixed. Elevated respiration necessarily must be due to oxygen use by the mitochondria and the corresponding production of CO_2_ as part of the process of generating energy rich ATP for use in cellular work. If male respiration rates were elevated in the selected lines, then it could represent a metabolic cost. 

 The energy source in the selection experiment was obtained by calculating the respiratory ratio (RQ). RQs were always approximately 1.0. The compounds metabolized for energy were carbohydrates. This is typical for Drosophila. There was nothing about the treatments or the response to selection that drove flies to use a different energy compound for metabolism in the present study. 

 A difference between sexes in the pattern of respiration at different assay stages was observed in the present study (Figure 13: per fly, Figure 14: adjusted by lean mass). The most consistent and pronounced effect was elevated respiration on day 7 after mating in females (lean mass, dry weight, or per fly). This could be due to females expending substantial energy to provision oocytes with yolk. Insulin signaling plays a critical role in the hormonal control of vitellogenesis [23]. Elevated insulin signaling could be a factor in the relationship between female mating and respiration rates observed in the present study. Differences in the sexes in the pattern of respiration rates at different assay stages might reflect fundamental differences between the physiology and reproductive biology of *D. melanogaster* females and males. The two sexes differ in many ways including their genome-wide patterns of gene expression [24].

**Figure 20:**
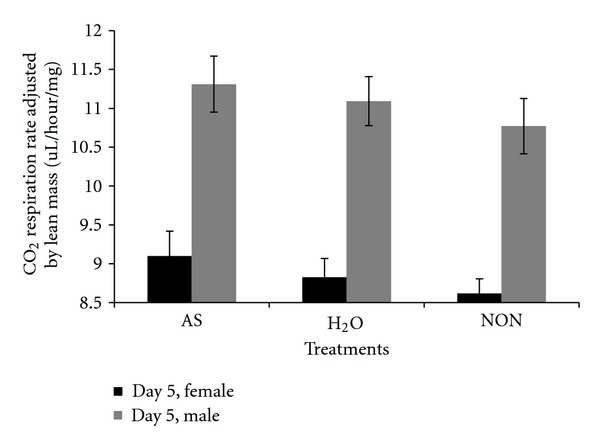
CO_2_ respiration rate on day 5 (after treatment) adjusted by lean mass for different treatments and each sex. AS: autoclaved spores, H_2_O: injected with sterile water as a wound control, NON: no perturbation.

**Figure 21:**
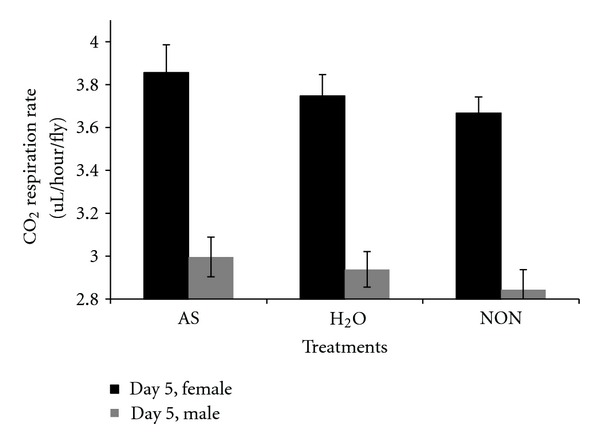
CO_2_ respiration rate on day 5 measured per fly for different treatments and each sex. AS: autoclaved spores, H_2_O: injected with sterile water as a wound control, NON: no perturbation.

**Figure 22:**
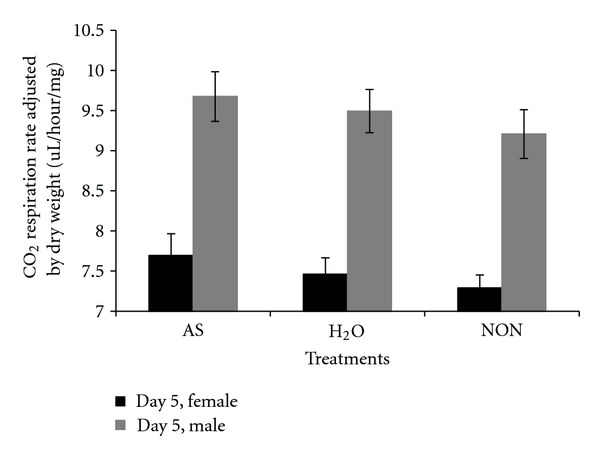
CO_2_ respiration rate on day 5 adjusted by dry weight for different treatments and each sex. AS: autoclaved spores, H_2_O: injected with sterile water as a wound control, NON: no perturbation.

 There were several issues concerning respiration rates that are pertinent to interpreting the results of the present study. One issue is whether to adjust respiration rate by dry weight as it includes stored lipid which is nonrespiring mass. Thus, biased data can be generated after adjusting by dry weight as illustrated by the following. As a general observation, *D. melanogaster* selected in the laboratory for stress resistance typically stores higher levels of lipid [18]. In previous studies, when respiration rate was measured for selection line flies, it appeared as though lower respiration rate was an indirect response to selection. However, when respiration rate was corrected for nonmetabolic mass (stored lipid) or reported per fly, then there was no reduction in respiration rate in the selected lines [18, 25]. In the present study, there were no differences among line types in lean mass, but there were in dry weight which suggests that adjusting respiration rates by dry weights could be biased by differences in the amount of stored lipid among line types. Another issue was whether there was evidence for a phenotypic response after flies were challenged by autoclaved spores. There was a trend indicating that male and female respiration rate was slightly elevated after flies were challenged by autoclaved spores relative to the other treatments even though there were no statistically significant differences (Figures 20, 21, and 22). Assuming that there was a small response to the spores, that was consistent over 19 generations of selection, the overall response to spores could have been amplified in the selected lines. Thus, a cumulative process could underlie any elevation in male respiration rate in the selected lines compared to the other lines. A final issue was whether day effects (age) could account for any of the respiration rate results. At the age (day 5) that treatments were administered, there was a set of flies that were not treated with autoclaved spores and were not wounded with a needle dipped in water. These untreated flies (NON) were controls for a day effect from day 3 to day 5. There was a statistically significant difference between day 3 and day 5 in respiration rate in flies adjusted by lean mass (*P* = 0.0044) whereby respiration rates dropped (Figure 14). However, it can be seen (Figure 20) that the untreated flies (NON) markedly dropped on day 5 indicating a day effect that was perhaps largely responsible for the reduced respiration rates in the other lines on this day. Two days later all flies were mated and there was no set of flies that remained unmated that could act as a control for day effects. In wild-type flies, respiration rate changes slowly as a function of age and the change is a gradual decrease [26, 27]. This is opposite of the response observed in the present study. Thus, the elevated respiration rates observed after mating on day 7 is not likely to be due to a day effect. 

### 4.3. Movement

 Movement was measured in the present study in an assay that soon followed the respiration measurements. There was a phenotypic difference between sexes, but no difference among lines or treatments. The absence of male movement differences between the selected and control lines indicates that increased movement could not have been responsible for increased respiration in selected line males as potentially documented in the present study. This inference is strongly supported by the experimental design as the movement assays were conducted on the same flies as used for respiration. Relatively elevated male *D. melanogaster* movement has been observed in another study [28]. In the present study there was a general trend across lines for males to move more than females. It is possible that males are more active in order to find mates and court, whereas females are relatively stationary as oviposition-related behaviors might require less movement than mate-seeking and related behaviors.

## Supplementary Material

The supplementary materials consist of statistical analyses, figures and a synopsis of mean respiration rates. The statistical reported are likelihood analyses of CO_2_ respiration rates and respiration rates adjusted by lean mass, dry weight and per fly. The presented statistics also includes respiration rates, post-hoc analyses and P values (S1A, S1B, S1C and S1D). One group of figures presents CO_2_ respiration rates for the three lines within each line type (S2A, S2B, S2C and S2D). A line figure presents CO_2_ respiration rates adjusted by lean mass and dry weight for female and males (S3). The data synopsis presents average (SE) CO_2_ respiration for all line types, assay stages, treatments and both sexes adjusted by lean mass (S4A), per fly (S4B) and adjusted by dry weight (S4C).

## Figures and Tables

**Table 1:** Designations of lines, treatments, and assay stages.

	Identifiers and description
Line types	S: selected with live spores, CP: wound control lines punctured with sterile H_2_O, CN: no perturbation
Treatments	AS: punctured with autoclaved (dead) spores, H_2_O: punctured with sterile H_2_O, NON: no treatment
Assay stages	Day 3: before perturbation, Day 5: punctured with autoclaved spores or punctured with sterile H_2_O or remained unperturbed, Day 7: after mating

**Table:** (a)

Assay stages	Lines	Treatments	Mean RQ	S.E.
Day 3	CP	AS	1.02	0.022
Day3	CP	NON	1.04	0.025
Day 3	CP	H_2_O	1.11	0.042
Day 3	CN	AS	1.06	0.029
Day 3	CN	NON	1.02	0.026
Day 3	CN	H_2_O	1.00	0.023
Day 3	S	AS	1.04	0.027
Day 3	S	NON	1.05	0.027
Day 3	S	H_2_O	1.07	0.025
Day 5	CP	AS	1.04	0.029
Day 5	CP	NON	0.97	0.022
Day 5	CP	H_2_O	1.04	0.035
Day 5	CN	AS	1.03	0.027
Day 5	CN	NON	0.99	0.023
Day 5	CN	H_2_O	0.98	0.019
Day 5	S	AS	1.00	0.033
Day 5	S	NON	1.03	0.032
Day 5	S	H_2_O	1.04	0.021
Day 7	CP	AS	1.03	0.031
Day 7	CP	NON	1.03	0.031
Day 7	CP	H_2_O	1.03	0.029
Day 7	CN	AS	1.05	0.027
Day 7	CN	NON	1.02	0.034
Day 7	CN	H_2_O	1.00	0.035
Day 7	S	AS	0.99	0.028
Day 7	S	NON	0.99	0.024
Day 7	S	H_2_O	1.03	0.026

**Table:** (b)

Assay stages	Lines	Treatments	Mean RQ	S.E.
Day 3	CI	AS	1.16	0.042
Day 3	CI	NON	1.10	0.030
Day 3	CI	H_2_O	1.11	0.028
Day 3	CN	AS	1.08	0.032
Day 3	CN	NON	1.13	0.032
Day 3	CN	H_2_O	1.08	0.033
Day 3	S	AS	1.11	0.031
Day 3	S	NON	1.09	0.035
Day 3	S	H_2_O	1.11	0.032
Day 5	CI	AS	1.10	0.040
Day 5	CI	NON	1.11	0.045
Day 5	CI	H_2_O	1.08	0.040
Day 5	CN	AS	1.10	0.032
Day 5	CN	NON	1.05	0.032
Day 5	CN	H_2_O	1.06	0.028
Day 5	S	AS	1.13	0.037
Day 5	S	NON	1.11	0.045
Day 5	S	H_2_O	1.12	0.030
Day 7	CI	AS	1.17	0.058
Day 7	CI	NON	1.10	0.046
Day 7	CI	H_2_O	1.06	0.038
Day 7	CN	AS	1.07	0.034
Day 7	CN	NON	1.10	0.033
Day 7	CN	H_2_O	1.10	0.047
Day 7	S	AS	1.07	0.035
Day 7	S	NON	1.13	0.056
Day 7	S	H_2_O	1.10	0.032
