# Supplementary material for: Drosophila melanogaster Selection for Survival after Infection with Bacillus cereus Spores: Evolutionary Genetic and Phenotypic Investigations of Respiration and Movement
Source: Int J Evol Biol. 2013 Mar 21;2013:576452. doi: 10.1155/2013/576452 (PMC3619139; doi:10.1155/2013/576452)
Supplement: Supplementary file 1 — The supplementary materials consist of statistical analyses, figures and a synopsis of mean respiration rates. The statistical reported are likelihood analyses of CO2 respiration rates and respiration rates adjusted by lean mass, dry weight and per fly. The presented statistics also includes respiration rates, post-hoc analyses and P values (S1A, S1B, S1C and S1D). One group of figures presents CO2 respiration rates for the three lines within each line type (S2A, S2B, S2C and S2D). A line figure presents CO2 respiration rates adjusted by lean mass and dry weight for female and males (S3). The data synopsis presents average (SE) CO2 respiration for all line types, assay stages, treatments and both sexes adjusted by lean mass (S4A), per fly (S4B) and adjusted by dry weight (S4C). [file 576452.f1.pdf]

S1A. Values of -2 Res Log Likelihood of respiration rates analysis

	-2 Res Log Likelihood	
	Line-to-Line Random Effect	No Random Effect
Male CO_2 Respiration Rate (ul/hour/fly)	2682.2	2682.2*
Female CO_2 Respiration Rate (ul/hour/fly)	3174.3*	3186.6
Male CO_2 Respiration Rate _Dry Weight (ul/hour/mg)	4929.3*	4939.9
Female CO_2 Respiration Rate _Dry Weight (ul/hour/mg)	4517.0*	4568.3
Male CO_2 Respiration Rate _Lean Mass (ul/hour/mg)	5230.0*	5237.8
Female CO_2 Respiration Rate _Lean Mass (ul/hour/mg)	4845.8*	4898.9

* designates models with better fit by either adding the random effect or not, which was estimated by comparing *Δ -2* log-likelihood* to $\chi^2_{(df=1)}$.

S1B. P-value of statistical analysis of CO_2 respiration rate adjusted by lean mass, determined per fly, or adjusted by dry weight for both males and females.

	P-value of CO_2 Respiration Rate Analysis					
	Lean Mass		Dry Weight		Per Fly	
	Male	Female	Male	Female	Male	Female
Line Type	0.4322	0.9856	0.8117	0.9597	0.0132	0.8339
Assay Stage	0.0005	<.0001	0.0046	<.0001	0.0087	<.0001
Treatment	0.2797	0.7246	0.2961	0.7274	0.3177	0.6804
Line Type*Treatment	0.6589	0.9777	0.6432	0.9792	0.6565	0.9789
Line Type*Assay Stage	0.6416	0.5711	0.4833	0.4112	0.5915	0.9658
Assay Stage*Treatment	0.3092	0.3973	0.3100	0.4125	0.3312	0.4200

S1C. P-value of post-hoc analysis of CO_2 respiration rate adjusted by lean mass, determined per fly, or adjusted by dry weight for both males and females based on the statistically significant differences observed in S1B.

	Adjusted P-value of CO_2 Respiration Rate Post-hoc Analysis					
	Lean Mass		Dry Weight		Per Fly	
	Male	Female	Male	Female	Male	Female
Day7 vs. Day5	0.0011	<.0001	0.0067	<.0001	0.4480	<.0001
Day7 vs. Day 3	0.9074	<.0001	0.8892	<.0001	0.0064	0.1049
Day5 vs. Day 3	0.0044	0.1361	0.0250	0.1506	0.1548	0.0624
CP vs. CN	-	-	-	-	0.9303	-
CP vs. S	-	-	-	-	0.0186	-
CN vs. S	-	-	-	-	0.0494	-

S1D. P-value of statistical analysis of lean mass and dry weight for both males and females.

	P-value of Weight Analysis			
	Lean Mass		Dry Weight	
	Male	Female	Male	Female
Line Type	0.3050	0.0004	0.0334	<.0001
Assay Stage	<.0001	<.0001	<.0001	<.0001
Treatment	0.4210	0.5118	0.3011	0.4402
Line Type*Treatment	0.5053	0.8804	0.5177	0.9205
Line Type*Assay Stage	0.5696	0.2568	0.9661	0.1571
Assay Stage*Treatment	0.2691	0.2810	0.3124	0.1185

S2A.

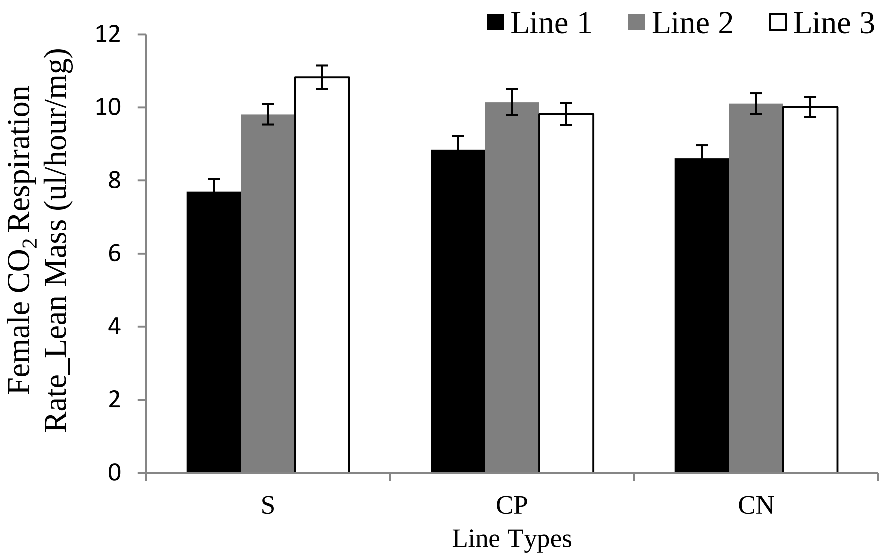

Line 1
Line 2
Line 3
Female CO_2 Respiration Rate_Lean Mass (ul/hour/mg)
12
10
8
6
4
2
0
S
CP
CN
Line Types

S2B.

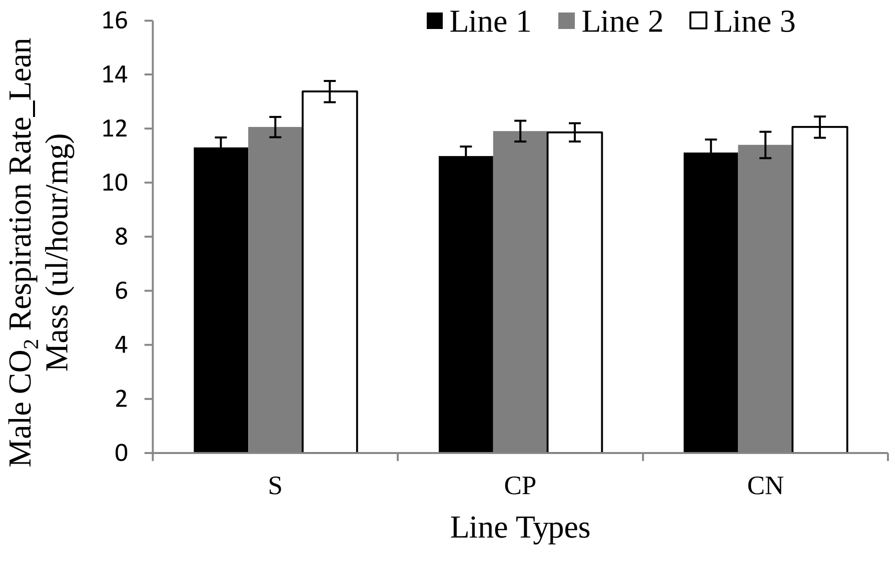

Line 1
Line 2
Line 3
Male CO_2 Respiration Rate_Lean Mass (ul/hour/mg)
16
14
12
10
8
6
4
2
0
S
CP
CN
Line Types

S2C.

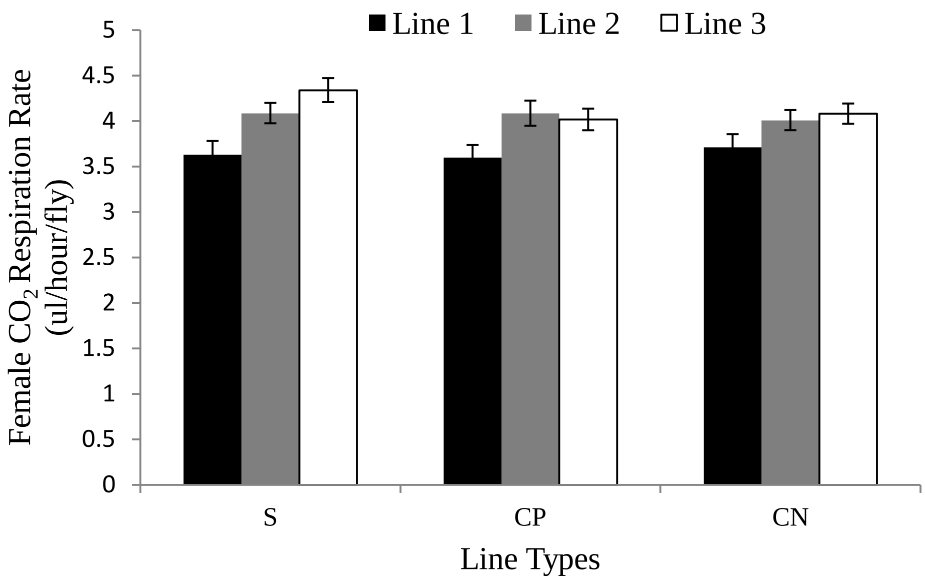
Line 1
Line 2
Line 3
5
4.5
4
3.5
3
2.5
2
1.5
1
0.5
0
Female CO_2 Respiration Rate
(ul/hour/fly)
S
CP
CN
Line Types

S2D

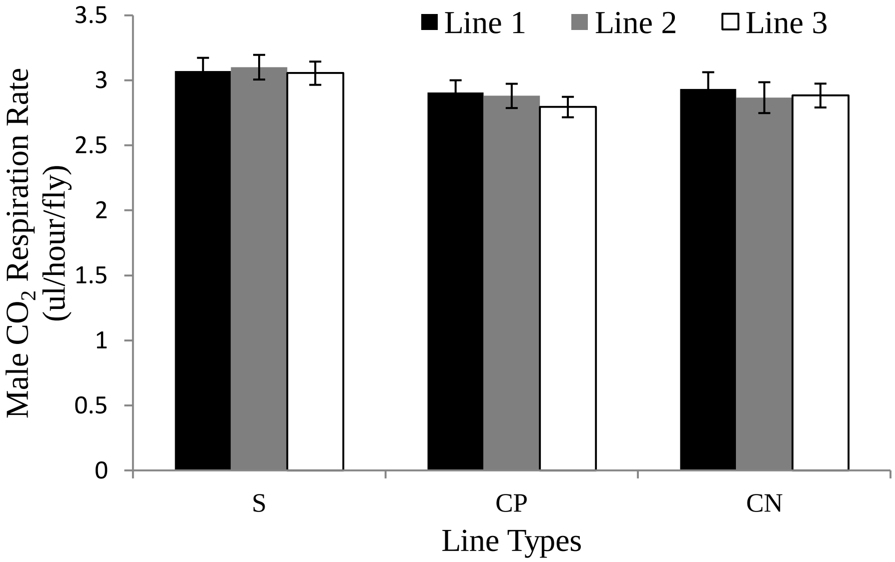
Line 1
Line 2
Line 3
3.5
3
2.5
2
1.5
1
0.5
0
Male CO_2 Respiration Rate
(ul/hour/fly)
S
CP
CN
Line Types

S3

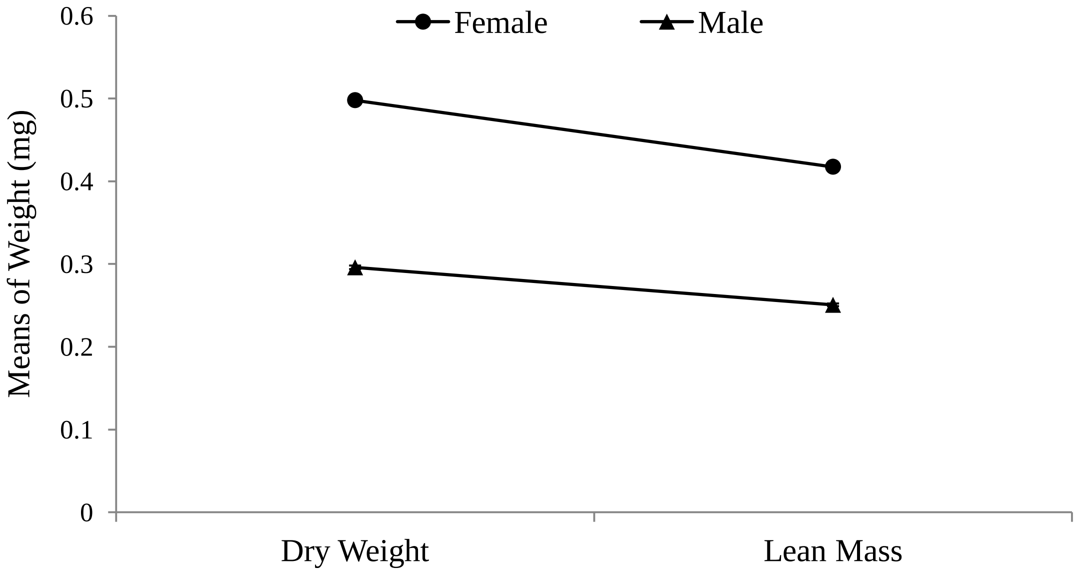

0.6
0.5
0.4
0.3
0.2
0.1
0
Means of Weight (mg)
Female
Male
Dry Weight
Lean Mass

S4A. Average CO_2 respiration rates adjusted by lean mass

Line Types	Assay Stages	Treatments	Respiration Rate Adjusted by Lean Mass (ul/hour/mg)			
			CO_2 _Female (SE)	O_2 _Female (SE)	CO_2 _Male (SE)	O_2 _Male (SE)
No Perturbation Control (CN)	Day 3	AS	9.72 (0.48)	9.28 (0.40)	11.45 (0.62)	10.85 (0.53)
No Perturbation Control (CN)	Day 3	H_2O	8.78 (0.34)	8.91 (0.31)	11.67 (0.88)	10.88 (0.68)
No Perturbation Control (CN)	Day 3	NON	9.02 (0.45)	9.04 (0.48)	12.75 (1.38)	11.72 (1.12)
No Perturbation Control (CN)	Day 5	AS	9.08 (0.50)	8.75 (0.37)	10.97 (0.52)	10.16 (0.51)
No Perturbation Control (CN)	Day 5	H_2O	9.24 (0.39)	9.41 (0.36)	10.50 (0.55)	10.03 (0.50)
No Perturbation Control (CN)	Day 5	NON	8.89 (0.38)	8.95 (0.31)	10.06 (0.34)	9.86 (0.42)
No Perturbation Control (CN)	Day 7	AS	9.84 (0.66)	9.58 (0.66)	11.49 (0.54)	11.00 (0.63)
No Perturbation Control (CN)	Day 7	H_2O	11.18 (0.75)	11.26 (0.75)	11.53 (0.52)	10.80 (0.46)
No Perturbation Control (CN)	Day 7	NON	10.42 (0.63)	10.47 (0.70)	13.16 (1.04)	11.81 (0.86)
Control Punctured (CP)	Day 3	AS	9.24 (0.39)	9.03 (0.32)	13.14 (0.65)	11.62 (0.55)
Control Punctured (CP)	Day 3	H_2O	9.80 (0.59)	9.51 (0.45)	10.48 (0.39)	10.22 (0.47)
Control Punctured (CP)	Day 3	NON	9.35 (0.52)	9.17 (0.45)	11.01(0.47)	10.22 (0.46)
Control Punctured (CP)	Day 5	AS	9.12 (0.62)	8.70 (0.44)	11.17 (0.69)	10.35 (0.59)
Control Punctured (CP)	Day 5	H_2O	8.20 (0.43)	8.12 (0.38)	11.02 (0.51)	10.34 (0.43)
Control Punctured (CP)	Day 5	NON	8.46 (0.24)	8.80 (0.26)	11.40 (0.94)	10.67 (0.89)
Control Punctured (CP)	Day 7	AS	10.65 (0.74)	10.65 (0.77)	11.53 (0.49)	10.23 (0.46)
Control Punctured (CP)	Day 7	H_2O	11.06 (0.79)	10.52 (0.79)	12.00 (0.67)	10.80 (0.53)
Control Punctured (CP)	Day 7	NON	10.80 (0.67)	10.66 (0.73)	12.39 (0.60)	11.52 (0.66)
Selected (S)	Day 3	AS	9.43 (0.59)	9.51 (0.47)	12.41 (0.79)	11.61 (0.54)
Selected (S)	Day 3	H_2O	9.02 (0.51)	8.79 (0.39)	12.79 (0.61)	11.85 (0.55)
Selected (S)	Day 3	NON	8.91 (0.58)	9.02 (0.49)	12.79 (0.82)	11.48 (0.60)
Selected (S)	Day 5	AS	9.10 (0.55)	8.94 (0.38)	11.75 (0.66)	10.53 (0.55)
Selected (S)	Day 5	H_2O	9.07 (0.42)	8.85 (0.40)	11.71 (0.56)	10.63 (0.51)
Selected (S)	Day 5	NON	8.50 (0.35)	8.39 (0.34)	10.89 (0.43)	10.17 (0.50)
Selected (S)	Day 7	AS	10.21 (0.62)	9.87 (0.67)	13.08 (0.99)	11.94 (0.89)
Selected (S)	Day 7	H_2O	10.31 (0.84)	9.78 (0.87)	11.75 (0.48)	10.82 (0.47)
Selected (S)	Day 7	NON	10.74 (0.64)	10.33 (0.67)	12.95 (0.58)	12.10 (0.58)

S4B. Average CO_2 respiration rates measured per fly

Line Types	Assay Stages	Treatments	Respiration Rate (ul/hour/fly)			
			CO_2 _Female (SE)	O_2 _Female (SE)	CO_2 _Male (SE)	O_2 _Male (SE)
No Perturbation Control (CN)	Day 3	AS	4.14 (0.21)	3.95 (0.18)	2.95 (0.16)	2.79 (0.14)
No Perturbation Control (CN)	Day 3	H_2O	3.73 (0.14)	3.78 (0.13)	3.00 (0.22)	2.80 (0.17)
No Perturbation Control (CN)	Day 3	NON	3.82 (0.19)	3.84 (0.20)	3.28 (0.36)	3/01 (0.29)
No Perturbation Control (CN)	Day 5	AS	3.76 (0.19)	3.63 (0.14)	2.89 (0.14)	2.67 (0.13)
No Perturbation Control (CN)	Day 5	H_2O	3.85 (0.15)	3.94 (0.15)	2.76 (0.15)	2.64 (0.13)
No Perturbation Control (CN)	Day 5	NON	3.71 (0.15)	3.74 (0.12)	2.65 (0.09)	2.60 (0.11)
No Perturbation Control (CN)	Day 7	AS	3.88 (0.26)	3.78 (0.26)	2.69 (0.13)	2.60 (0.16)
No Perturbation Control (CN)	Day 7	H_2O	4.41 (0.30)	4.44 (0.30)	2.72 (0.12)	2.56 (0.11)
No Perturbation Control (CN)	Day 7	NON	4.10 (0.25)	4.11 (0.27)	3.08 (0.23)	2.76 (0.20)
Control Punctured (CP)	Day 3	AS	3.83 (0.16)	3.75 (0.13)	3.34 (0.17)	3.00 (0.14)
Control Punctured (CP)	Day 3	H_2O	4.06 (0.24)	3.95 (0.19)	2.66 (0.10)	2.60 (0.12)
Control Punctured (CP)	Day 3	NON	3.87 (0.21)	3.80 (0.19)	2.80 (0.12)	2.60 (0.12)
Control Punctured (CP)	Day 5	AS	3.90 (0.26)	3.72 (0.18)	2.92 (0.18)	2.71 (0.16)
Control Punctured (CP)	Day 5	H_2O	3.50 (0.19)	3.47 (0.16)	2.85 (0.13)	2.68 (0.11)
Control Punctured (CP)	Day 5	NON	3.62 (0.10)	3.77 (0.11)	2.96 (0.25)	2.76 (0.23)
Control Punctured (CP)	Day 7	AS	4.07 (0.27)	4.07 (0.29)	2.63 (0.10)	2.33 (0.10)
Control Punctured (CP)	Day 7	H_2O	4.23 (0.30)	4.02 (0.30)	2.74 (0.15)	2.47 (0.12)
Control Punctured (CP)	Day 7	NON	4.12 (0.25)	4.06 (0.27)	2.83 (0.13)	2.62 (0.14)
Selected (S)	Day 3	AS	4.21 (0.24)	4.26 (0.19)	3.13 (0.20)	2.93 (0.13)
Selected (S)	Day 3	H_2O	4.01 (0.20)	3.91 (0.15)	3.22 (0.15)	2.99 (0.14)
Selected (S)	Day 3	NON	4.00 (0.23)	4.03 (0.20)	3.23 (0.22)	2.90 (0.16)
Selected (S)	Day 5	AS	3.92 (0.22)	3.87 (0.15)	3.17 (0.16)	2.84 (0.13)
Selected (S)	Day 5	H_2O	3.90 (0.18)	3.63 (0.16)	3.18 (0.15)	2.89 (0.14)
Selected (S)	Day 5	NON	3.67 (0.14)	3.79 (0.13)	2.93 (0.11)	2.73 (0.12)
Selected (S)	Day 7	AS	4.12 (0.25)	3.99 (0.27)	3.04 (0.21)	2.78 (0.19)
Selected (S)	Day 7	H_2O	4.13 (0.34)	3.91 (0.35)	2.73 (0.11)	2.51 (0.10)
Selected (S)	Day 7	NON	4.31 (0.25)	4.15 (0.27)	3.02 (0.14)	2.81 (0.13)

S4C. Average CO_2 respiration rates adjusted by dry weight

Line Types	Assay Stages	Treatments	Respiration Rate _ Dry Weight (ul/hour/mg)			
			CO_2 _Female (SE)	O_2 _Female (SE)	CO_2 _Male (SE)	O_2 _Male (SE)
No Perturbation Control (CN)	Day 3	AS	8.21 (0.43)	7.83 (0.34)	9.89 (0.54)	9.37 (0.46)
No Perturbation Control (CN)	Day 3	H_2O	7.41 (0.30)	7.51 (0.27)	10.09 (0.76)	9.41 (0.59)
No Perturbation Control (CN)	Day 3	NON	7.62 (0.39)	7.63 (0.41)	11.03 (1.19)	10.14 (0.98)
No Perturbation Control (CN)	Day 5	AS	7.72 (0.42)	7.44 (0.31)	9.41 (0.44)	8.71 (0.44)
No Perturbation Control (CN)	Day 5	H_2O	7.87 (0.33)	8.02 (0.31)	9.01 (0.47)	8.60 (0.42)
No Perturbation Control (CN)	Day 5	NON	7.57 (0.33)	7.61 (0.27)	8.61 (0.29)	8.44 (0.36)
No Perturbation Control (CN)	Day 7	AS	8.01 (0.54)	7.81 (0.54)	9.86 (0.48)	9.44 (0.55)
No Perturbation Control (CN)	Day 7	H_2O	9.10 (0.61)	9.17 (0.61)	9.92 (0.45)	9.30 (0.40)
No Perturbation Control (CN)	Day 7	NON	8.49 (0.51)	8.53 (0.57)	11.29 (0.90)	10.14 (0.75)
Control Punctured (CP)	Day 3	AS	7.81 (0.33)	7.64 (0.27)	11.10 (0.55)	9.81 (0.46)
Control Punctured (CP)	Day 3	H_2O	8.27 (0.50)	8.03 (0.38)	8.89 (0.35)	8.65 (0.41)
Control Punctured (CP)	Day 3	NON	7.91 (0.45)	7.76 (0.40)	9.30 (0.41)	8.63 (0.40)
Control Punctured (CP)	Day 5	AS	7.76 (0.53)	7.40 (0.37)	9.52 (0.59)	8.83 (0.51)
Control Punctured (CP)	Day 5	H_2O	6.97 (0.37)	6.90 (0.33)	9.41 (0.44)	8.82 (0.37)
Control Punctured (CP)	Day 5	NON	7.19 (0.21)	7.48 (0.22)	9.72 (0.80)	9.10 (0.76)
Control Punctured (CP)	Day 7	AS	8.89 (0.62)	8.89 (0.64)	9.85 (0.43)	8.73 (0.40)
Control Punctured (CP)	Day 7	H_2O	9.22 (0.67)	8.78 (0.66)	10.24 (0.60)	9.22 (0.48)
Control Punctured (CP)	Day 7	NON	9.02 (0.56)	8.90 (0.61)	10.54 (0.50)	9.80 (0.55)
Selected (S)	Day 3	AS	7.95 (0.49)	8.01 (0.39)	10.17 (0.66)	9.51 (0.45)
Selected (S)	Day 3	H_2O	7.60 (0.43)	7.40 (0.33)	10.47 (0.51)	9.71 (0.46)
Selected (S)	Day 3	NON	7.51 (0.49)	7.60 (0.41)	10.46 (0.66)	9.39 (0.48)
Selected (S)	Day 5	AS	7.60 (0.45)	7.48 (0.31)	10.05 (0.56)	9.01 (0.47)
Selected (S)	Day 5	H_2O	7.58 (0.35)	7.38 (0.33)	10.03 (0.48)	9.10 (0.43)
Selected (S)	Day 5	NON	7.10 (0.29)	7.01 (0.27)	9.32 (0.37)	8.70 (0.43)
Selected (S)	Day 7	AS	8.45 (0.51)	8.17 (0.55)	10.71 (0.78)	9.78 (0.71)
Selected (S)	Day 7	H_2O	8.51 (0.69)	8.08 (0.72)	9.63 (0.38)	8.88 (0.38)
Selected (S)	Day 7	NON	8.88 (0.52)	8.54 (0.55)	10.63 (0.48)	9.92 (0.47)
